# Longitudinal Patterns of the Tip-of-the-Tongue Phenomenon in People With Subjective Cognitive Complaints and Mild Cognitive Impairment

**DOI:** 10.3389/fpsyg.2020.00425

**Published:** 2020-03-13

**Authors:** María Campos-Magdaleno, David Leiva, Arturo X. Pereiro, Cristina Lojo-Seoane, Sabela C. Mallo, Ana Nieto-Vieites, Onésimo Juncos-Rabadán, David Facal

**Affiliations:** ^1^Department of Developmental Psychology, University of Santiago de Compostela, Galicia, Spain; ^2^Department of Social Psychology and Quantitative Psychology, Institute of Neurosciences, University of Barcelona, Barcelona, Spain

**Keywords:** tip-of-the-tongue, lexical access, mild cognitive impairment, linear mixed models, longitudinal study, Compostela Aging Study

## Abstract

**Background:**

The Tip-of-the-Tongue (ToTs) state is considered a universal phenomenon and is a frequent cognitive complaint in old age. Previous cross-sectional studies have found that ToT measures successfully discriminate between cognitively unimpaired adults and adults with Mild Cognitive Impairment (MCI). The aim of this study was to identify longitudinal patterns of ToTs in individuals with subjective complaints and with MCI regarding progress of their cognitive status.

**Method:**

The study included 193 participants with subjective cognitive complaints (SCC) and 56 participants with MCI who completed a baseline and two follow-up assessments, with an interval of about 18 months between each assessment. Participants were classified into three groups by considering cognitive stability or deterioration from the baseline diagnosis: SCC-stable, MCI-stable and MCI-worsened. Participants performed a ToT task involving recognition and naming of famous people depicted in 50 photographs. Generalized Linear Mixed Models (GLMM) were used to model longitudinal changes in familiarity, feeling of knowing, semantic access, phonological access and verbal fluency.

**Results:**

Phonological access differentiated MCI patients, stable and worsened, from adults with SCCs at all evaluation times. Phonological access declined over time in the three groups, without significant interactions between groups and time.

**Discussion:**

This study provides the first longitudinal evidence of differences in ToT measures for adults with MCI. The findings indicate that phonological access measures successfully differentiated between the diagnostic groups. However, slopes remain irrespective of the diagnostic group and progression toward more advance stages of cognitive impairment.

## Introduction

Cognitive impairment in old adults has been considered a continuum including different stages (Jack et al., 2018): a cognitively unimpaired phase (CU), with performance within the expected range for age and education; presence of subjective cognitive complaints (SCC), without objective cognitive impairment (Jessen et al., 2014; Molinuevo et al., 2017); Mild Cognitive Impairment (MCI), characterized by the presence of cognitive complaints, objective mild cognitive deterioration and relative preservation of instrumental activities of daily living (Petersen, 2004; Petersen et al., 2018); and dementia or major neurocognitive disorder, characterized by cognitive affectation and psychological symptoms that cause dependency (American Psychiatric Association, 2013). In MCI, single and multiple domain subtypes (deterioration in only one or in more cognitive domains) have been used to describe different degrees of severity, with the multiple domain subtype being the most serious condition (Brambati et al., 2009; Han et al., 2012; Campos-Magdaleno et al., 2016). Progression into this continuum is a complex process characterized by cognitive changes, transitions and diagnostic instability, with an increased risk of conversion to dementia but also the possibility of regression to CU (Facal et al., 2015; Petersen et al., 2018). MCI entity is heterogeneous, and different subtypes according evolutionary trajectories and severity need to be addressed (Díaz-Mardomingo et al., 2017).

Language measures such as verbal fluency, naming and word learning have been successfully used as predictors of MCI and its progression to dementia (Murphy et al., 2006; Clague et al., 2011; Campos-Magdaleno et al., 2017). Tip-of-the-Tongue (ToT) constitutes one of the most frequent age-related language complaints and is characterized as a strong feeling of knowing in parallel with an inability to recall a lexical item which is known and that might eventually be recalled if enough attention and encoding feedback is provided (Brown, 2012; Bloom et al., 2018). Age-related increases in ToT experiences (hereafter ToTs) are not related to increased vocabulary knowledge throughout adulthood (Facal et al., 2012; Salthouse and Mendell, 2013; Shafto et al., 2017). Consistent evidence supports the hypothesis that the higher frequency of ToTs in older adults is caused by a decline in transmission of the activation from semantic to phonological representations (Burke et al., 1991; James and Burke, 2000; Shafto et al., 2007; Juncos-Rabadán et al., 2010; White et al., 2013). According to this hypothesis, ToTs occur when the activated semantic representation of a word fails to spread the necessary activation to its corresponding phonological representation, making lexical access impossible. The increase in the frequency of ToTs in older adults is consistent with an age-related decline in activation transmission, and proper names seem to be more vulnerable to this decline than common nouns, as proper names are represented by the individual characteristics of a person rather than by more general information connected to multiple semantic nodes (Burke et al., 1991). Other relevant hypothesis on cognitive aging, such as the inhibition deficit, explained ToT as a deficient inhibition of different phonological representations (competitors) that arise when semantic representation of the target word is successfully activated (Woodworth, 1938). However, few experimental studies (Jones and Langford, 1987; Jones, 1989) have supported that hypothesis, and other studies have not been able to replicate them (Meyer and Bock, 1992; Perfect and Hanley, 1992).

According to the cognitive continuum between unimpaired cognition and dementia, MCI represents an intermediate stage in the ability to retrieve proper names and is characterized by greater difficulty in phonological access, relative to cognitively unimpaired old adults, and only mild difficulties in semantic access more commonly associated with the onset of Alzheimer’s disease (Juncos-Rabadán et al., 2014). Several ToT measures, including semantic access (calculated as a proportional measure that represents successful access in the total number of target names) and phonological access (calculated as the proportion of successful semantic retrievals in which success in phonological access is also achieved) (Gollan and Brown, 2006; Juncos-Rabadán et al., 2010), have been successfully used as language predictors of MCI (Juncos-Rabadán et al., 2013). A multivariate logistic regression model including feeling of knowing, semantic knowledge, semantic access and phonological access was used to assess the predictive value of ToT measures for discriminating between normal controls and MCI patients within the Compostela Aging Study (CompAS). In a cross-sectional study, Juncos-Rabadán et al. (2013) found that a model including these four ToT measures together correctly classified 70% of controls (specificity) and 71.6 of MCI patients (sensitivity), with an Area Under Curve Roc (AUC) value of 0.74, and accounted for 23.5% of the variance. Although the model comprised all ToT variables, only the phonological access measure remained significantly associated with amnestic MCI. The authors also found that specificity, sensitivity and AUC values were higher than those obtained using semantic fluency as a language measure to discriminate MCI (total classification value, AUC = 0.66 and accounted variance = 15.4%). We have to mention two studies (Poppe et al., 2006; Oh and Ha, 2015) that did not find differences between normal oldest people and MCI patients, but they used the percent or the total number of produced ToTs that have been criticized as not appropriate measures because they do not explain the semantic and phonological representation and processes involved in ToT (Gollan and Brown, 2006).

Longitudinal studies on ToTs in MCI are very scarce. As far as we know, apart of the aforementioned by Poppe et al. (2006) that used the total number of reported ToTs, only one follow-up study of changes in ToT in MCI has been carried out to date (Facal et al., 2016a). In the aforementioned study, proportional measures of change between baseline and one follow-up assessment (around 18 months) were calculated for familiarity, semantic access, phonological access and semantic fluency in a sample of 15 individuals with multiple domain amnestic MCI, 41 individuals with single domain amnestic MCI and 41 cognitively unimpaired controls. Comparisons revealed significant differences between baseline and follow-up only in semantic and phonological access, with improvements in semantic access in the control group and decline in phonological access in the two groups with amnestic MCI. Nevertheless, full longitudinal models have been used to study change in semantic and phonological access and their potential role in explaining diagnostic change in MCI.

Facal et al. (2016a) also considers “familiarity” as a ToT measure of meta-cognitive processes involved in ToTs that indicate that the name knowledge is present (Schwartz and Metcalfe, 2011). Although some cross-sectional studies suggest that familiarity-based memory measures may be sensitive markers of preclinical and prodromal Alzheimer’s Disease (AD, Wolk et al., 2013; Pitarque et al., 2016), the longitudinal approach did not show any evidence of their predictive value (Facal et al., 2016a).

The aim of the present study was to determine longitudinal patterns of several ToT measures (mainly semantic and phonological access) by using linear mixed models and data from longitudinally assessed individuals with SCC and MCI classified on the basis of diagnostic stability or deterioration. With this objective we expected to obtain new evidence regarding the usefulness of these measures as linguistic markers to characterize the cognitive profile of adults with MCI.

## Materials and Methods

### Participants

Two hundred forty-nine adults in the range of 50–87 years old already participating in the Compostela Aging Study (CompAS) and who completed 3 extensive clinical and neuropsychological assessments (Baseline, Time 1, and Time 2) were included in this study. At baseline there were 407 participants who performed the ToTs tasks, but only 249 completed the 3 assessments, being the total rate of attrition around 38% (158 participants) due to motivation, mobility or morbidity. CompAS is an ongoing longitudinal project (Juncos-Rabadán et al., 2012) in which participants are recruited after referral by general practitioners from primary care centers in Galicia (an autonomous region in north-western Spain) subjective cognitive complaints. A study on the attrition in the general CompAS project and their raisons may be see in Facal et al. (2016b). Exclusion criteria included previous diagnosis of any neurological or psychiatric disease, dementia, MCI, clinical stroke, motor-sensory defects, alcohol or drug abuse/dependency and traumatic brain injury at baseline. All participants underwent the same extensive assessment, and were classified into SCC or MCI groups at a special meeting of the research team. MCI subjects were classified into four subtypes following standard criteria (Petersen, 2004; Dubois et al., 2007; Albert et al., 2011): single-domain amnestic MCI (sda-MCI); multiple-domain amnestic MCI (mda-MCI); single-domain non-amnestic MCI (sdna-MCI); and multiple-domain non-amnestic MCI (mdna-MCI). All MCI participants fulfilled the general criteria outlined by the National Institute on Aging-Alzheimer’s Association (Albert et al., 2011): (a) informant-corroborated memory complaints, assessed by a short version of the Subjective Memory Complaints Questionnaire (SMCQ; Benedet and Seisdedos, 1996); (b) performance of 1.5 standard deviations below age and education norms in at least one cognitive domain, assessed by the subscales of the Spanish-adapted version of the Cambridge Cognitive Examination (CAMCOG-R, Huppert et al., 1996; Spanish version: López-Pousa, 2003; Pereiro et al., 2015), apart from the memory domain, which was assessed by the Short and Long Delay Free Recall from the Spanish-adapted version of the California Verbal Learning Test (CVLT, Delis et al., 1987; Spanish version: Benedet and Alejandre, 1998); (c) no significant impact on activities of daily living, assessed by the Lawton and Brody Index (Lawton and Brody, 1969); and (d) no dementia, according the National Institute of Neurological and Communicative Disorders and Stroke-Alzheimer’s Disease and Related Disorders Association (NINCDS-ADRDA), and the Diagnostic and Statistical Manual of Mental Disorders- Fourth Edition (DSM-IV) criteria. Participants were classified as SCC when, presenting subjective cognitive complains to their general practitioners confirmed by their own responses and that from their relatives to the SMCQ, they performed as cognitively unimpaired adults according to norms for age and years of education in general functioning and specific domain tests assessed with CAMCOG-R and the CVLT.

All participants and their proxies were informed of the longitudinal nature of the project and were contacted twice regarding participation in two successive follow-up assessments with an interval of 18.67 ± 2.73 months between each assessment. This time interval maximizes participation and motivation, while and reduces attrition due morbidity, mobility and mortality (Facal et al., 2016b). After the second follow-up assessment, participants were classified into three groups by considering stability or progression from the diagnostic established at baseline: SCC participants at Baseline assessment who remained stable at Time 2 follow-up (SCC-Stable group, *n* = 193, 77.52%, 136 women/56 men); MCI participants at Baseline assessment who remained stable at Time 2 follow-up (MCI-Stable group, *n* = 33, 13.24%, 20 women/13 men); and sda-MCI or sdna-MCI participants at Baseline assessment who had progressed to mda-MCI, mdna-MCI or dementia either at Time 1 or Time 2 follow-up evaluations (MCI-Worsened group, *n* = 23, 9.24%, 16 women/7 men). Differences in the groups size reflect the incidence of MCI in people with subjective cognitive complains who attend primary care centers and the different rates of stability or progression/worsening (Facal et al., 2019).

Assessment of participants who progressed to probable AD or dementia was conducted according to the DMS-IV and NINCDS-ADRDA criteria, by checking the medical history and recording the date of neurological diagnosis.

All participants gave their written informed consent prior to participation in the study. The research project was approved by the Galician Clinical Research Ethics Committee (Xunta de Galicia, Spain), and the study was performed in accordance with the ethical standards established in the Declaration of Helsinki, updated in Seoul in 2008.

### Materials and Procedure

The target items were 50 color photographs of famous people of the last 50 years (actors, singers, politicians, sportsmen, arts personalities, etc. from Spain and other countries, see Supplementary Material 1) selected from a set of 70. They were previously presented to a small control group of cognitively unimpaired users (20 persons) of a life-long learning association from Santiago de Compostela (ATEGAL) aged between 55 and 80 years. Final 50 photographs correspond with those that obtained the highest punctuation in familiarity and semantic information (age, residence, marital status… of the celebrity), in order to maximize the probability of ToT states.

**FIGURE 1:**
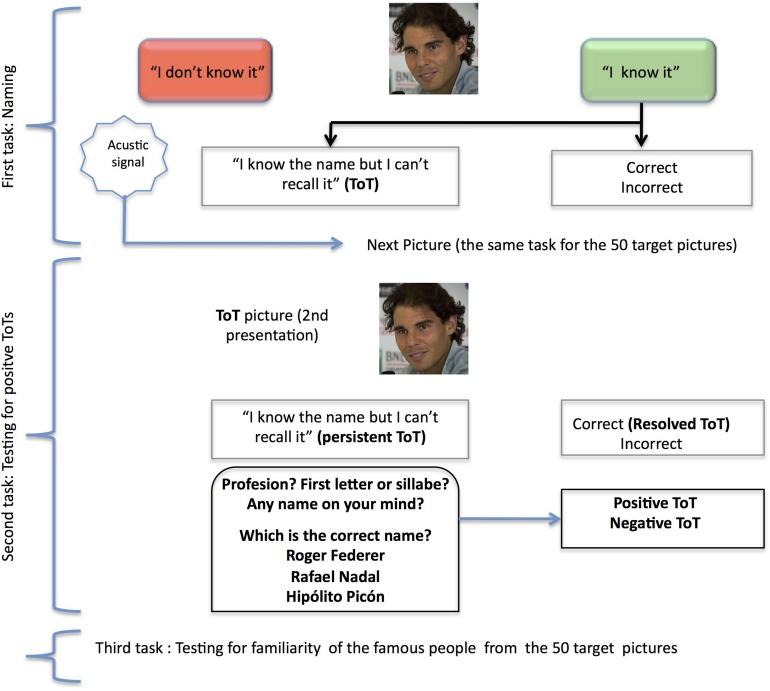
Representation of the tasks included in the ToT procedure. The photograph shown in the example is of Rafael Nadal, a famous Spanish tennis player. Photography by Valentina Alemanno (CC), modified according to figure size requirements from https://www.flickr.com/photos/thevhale/14309864633.

The ToT procedure included in the CompAS has been described in detail in a previous study (Juncos-Rabadán et al., 2011). In brief, the ToT procedure consisted of three tasks: (i) a naming task; (ii) a task to determine whether the ToTs were positive (when the name on the ToT was indeed the correct name) or negative (when the name on the ToT was not the target name); and (iii) a familiarity task, to assess the subjective degree of knowledge that each participant declared having about each celebrity depicted in the photographs (see Figure 1). In the naming task, 50 photographs of celebrities were presented separately on a screen (with E-Prime for Windows). Participants were asked to press the green key on a response box if they knew the name and the red key if they did not know the name. They were also asked to say the name out loud or to say either “I don’t know the name” or “I can’t recall the name at the moment” at the same time as pressing the response key. The names and responses were registered as follows: (a) correct (CORs) or incorrect, according to the accuracy of the name; (b) “Don’t know,” when the participant did not know the name; and (c) ToT state, when the participant said that they knew the name but could not recall it at the moment. In the second phase, the photographs that produced ToT responses were presented in a second task, in which participants were again asked for the celebrity’s name. If the participant correctly produced the name during the task, the response was classified as a resolved ToT. When the ToT was maintained or an incorrect name was produced, participants were encouraged to answer several questions that appeared on the screen in order to test their knowledge about the person and their name: ‘What is the person’s profession?’, ‘What is the first letter or syllable of the name?’, ‘Does any name come to your mind?’. After these questions were scored, the target name was presented with two non-target names. For each such triad, participants were asked to state which of the names presented separately on the screen was the correct name of the person in the previously presented photograph and if it was the name that they had been trying to remember when they said “I know the name but I can’t recall it.” The ToT was then classified as a positive ToT (pToT) when participants correctly recognized the target name and said that it was the name that they had been trying to remember, and negative ToT when they recognized it but said that it was not name on their mind. In the third phase, the 50 target pictures were presented to each participant to determine how familiar the famous people were. Responses were scored on a scale of 1–5 (where 5 represents maximum familiarity and 1, unfamiliarity).

The following measures were considered for the purposes of this study: (A) Familiarity, which represents the subjective knowledge that participants had about the people represented in the target pictures. This was calculated by summing the familiarity responses for all 50 photographs. (B) Feeling of Knowing, which represents the security that participants have about the knowing the name, independently of whether the name was recalled or not (Schwartz, 2002). This was measured as the number of times that participants pressed the green key in the naming task. (C) Semantic access was calculated by the equation [(CORs + pToTs)/N] and represents successful access to the semantic representations of the names. (D) Phonological access was calculated by the equation [CORs/(CORs + pToTs)] and represents the proportion of both successful semantic and phonological retrievals (Juncos-Rabadán et al., 2010).

In addition to these ToT measures, two lexical measures were considered: (A) Verbal fluency-animals (Semantic fluency), defined as the ability to produce words within a fixed time interval (Lezak et al., 2004) and considered suitable for detecting MCI (Taler and Phillips, 2009), was used as a general measure of lexical access; and (B) Total scoring in the vocabulary test of the Wechsler Adult Intelligence Scale (WAIS; Wechsler, 1988), used to measure the general verbal knowledge of the participant.

### Statistical Analysis

Considering the heterogeneity in the sample size of the groups, non-parametric tests (e.g., Kruskal–Wallis and Mann–Whitney tests) were used to analyze between-group differences in socio-demographic and ToT measures at baseline. Complementarily, parametric tests were included also analyzing between-group differences. We initially selected Generalized Linear Mixed Models (GLMM) for modeling longitudinal changes in the language, including random intercepts and random slopes. Thus, patterns of performance in lexical access can be represented by different slopes and longitudinal trajectories can be defined by the intercepts. However, and due to convergence problems, random slopes were excluded from the analyses.

We created the statistical models including Evaluation Time (Baseline, Time 1, and Time 2), Group (SCC-Stable, MCI-Stable and MCI-Worsened), and the interactions (Evaluation Time × Group) as independent variables or predictors as fixed effects. Pairwise comparisons of the estimated marginal means for the dependent variables Evaluation Time and Group was carried out after they were specified as factors. We included heteroskedasticity due to group, random effects for intercepts, and the covariates age at baseline and previously standardized vocabulary score in all models (see Supplementary Material 2).

Separate models were obtained for each dependent variable, with SCC-Stable as the reference group and Baseline assessment as the reference evaluation time. LMMs assuming a Gaussian response were used for modeling changes in proportional measures. GLMMs assuming Poissonian response were selected for counting measures. When statistical assumptions (e.g., overdispersion of data) were not met in the GLMMs, a negative binomial distribution was used for modeling count data.

Log Likelihood, Akaike and Bayesian Information Criteria indices of goodness of fit were used to select the best models for each response. Thus, in order to select the best model for predicting the intercepts and slopes in each group by the ToT measures, we first compared all possible models including fixed effects (i.e., Evaluation time, Group and their interaction) and including random effects or not. After optimizing the structure for the fixed and random effects, we then added heteroskedasticity (between-group variability) to the model and chose the best-fit model. Finally, we included standardized covariates in the model to allow intercept interpretation (see Supplementary Material 2 to reproduce the steps to get these intermediate models as well as the final regression models hereby detailed).

Cross-sectional statistical analysis was performed with SPSS for Windows, version 21.0 (SPSS, Chicago, IL, United States); (G)LMMs were estimated in R environment (version 3.5.3; R Core Team, 2019) with the nlme (version 3.1-1137; Pinheiro et al., 2018) and lme4 packages (version 1.1-21; Bates et al., 2015).

## Results

Socio-demographic, neuropsychological and ToT measures of the groups at baseline are summarized in Table 1. The MCI-Worsened group was the oldest, followed by MCI-Stable. The SCC-Stable group obtained higher scores than the two MCI groups at baseline for the cognitive measures, MiniMental State Examination (MMSE; Folstein et al., 1975; Spanish version: Lobo et al., 1999) and CLVT Short (CVLT-SDFR) and Long Delay Free Recall (CVLT-LDFR), as well as for the TOT measures, semantic access and phonological access and semantic fluency. Familiarity was higher in the SCC-Stable and MCI-Stable groups than in the MCI-Worsened group. Vocabulary level was highest for the SCC-Stable than the other two groups. The lowest feeling of knowing was obtained in the MCI-Worsened group. No differences were found at baseline in comorbidity, and differences in years of education were only obtained between the SCC Stable and MCI Worsened group in the parametric comparisons.

**TABLE 1:** Mean values and standard deviations (in parentheses) of the demographic, cognitive and TOT measures by the three cognitively normal groups: subjective cognitive complaints (SCC) that remained Stable (SCC Stable), Mild Cognitive Impairment that remained Stable (MCI Stable), and MCI that worsened (MCI Worsened).

	SCC Stable Group 1 *N* = 193	MCI Stable Group 2 *N* = 33	MCI Worsened Group 3 *N* = 23	Group differences -Kruskal–Wallis χ^2^(gl)	Groups comparison – Mann–Whitney tests	Groups differences-ANOVAs *F*(2,146)	Groups comparison Bonferroni tests
Age	64.94 (8.84) Range: 50–87	69.27 (7.35) Range: 54–82	73.91 (7.15) Range: 61–87	24.15 (2)**	Group3 > Group2 > Group1	41.66**	Group3 > Group2 > Group1
Years Education	10.18 (4.66) Range: 2–22	9.06 (4.58) Range: 3–21	8.35 (3.70) Range: 2–18	4.51 (2)		6.64*	Group3 < Group1
CCI	0.87 (0.89) Range: 0–3	0.75 (−93) Range: 0-4	1.04 (0.97) Range: 0–3	1.56 (2)		0.68	
Lawton	7.60 (0.88) Range: 4–8	6.84 (1.40) Range: 3–8	6.55 (1.93) Range: 3–8	16.33 (2)**	Group3, Group2 < Group1	11.80**	Group3 < Group2, Group1
SCC- Patient	18.66 (4.34) Range: 7–32	20.22 (4.21) Range: 10–29	19.47 (4.45) Range: 13–33	7.09 (2) *	Group2 > Group1	2.02	
SCC-Informant	15.59 (4.49) Range: 2–29	16.53 (3.72) Range: 9–22	18.25 (4.31) Range: 12–26	6.83 (2)*	Group3 > Group1	3.78*	Group3 > Group1
MMSE	28.16 (1.64) Range: 21–30	25.75 (2.63) Range: 21–30	25.17 (2.53) Range: 19–29	50.82 (2)**	Group3, Group2 < Group1	42.62**	Group3, Group2 < Group1
CVLT-SDFR	10.64 (2.71) Range: 3–16	4.96 (3.15) Range: 0–11	4.43 (3.08) Range: 0–11	87.23 (2)**	Group3, Group2 < Group1	96.77**	Group3, Group2 < Group1
CVLT-LDFR	11.44 (2.91) Range: 3–16	6.27 (3.91) Range: 0–14	4.82 (2.99) Range: 0–11	79.95 (2)**	Group3, Group2 < Group1	78.25**	Group3, Group2 < Group1
WAIS vocabulary subscale	51.18 (13.07) Range: 19–75	41.27 (13.00) Range: 20–71	41.48 (14.47) Range: 15–64	20.89 (2)**	Group3, Group2 < Group1	36.43**	Group3, Group2 < Group1
Semantic access proportion	0.92 (0.09) Range:0.40–1	0.87 (0.12) Range:0.40–1	0.74 (0.20) Range: 0.22–1	15.97 (2)**	Group3, Group2 < Group1	70.54**	Group3 < Group2 < Group1
Phonological access proportion	0.82 (0.13) Range:0.09 – 1	0.66 (0.20) Range:0.08–1	0.62 (0.61) Range:0.18–0.98	13.95 (2)**	Group3, Group2 < Group1	70.37**	Group3, Group2 < Group1
Familiarity	222.87 (28–28) Range: 89–250	223.44 (27.96) Range: 127–250	204.14 (36.42) Range: 116–250	16.98 (2)**	Group3 < Group2, Group1	10.78**	Group3 < Group2, Group1
Feeling of Knowing	48.05 (3.57) Range: 23–50	46.75 (4.64) Range: 24–50	41.40 (9.53) Range: 17–50	45.29 (2)**	Group3 < Group2 < Group1	51.79**	Group3 < Group2, Group1
Semantic fluency	17.75 (5.79) Range: 6–35	13.66 (4.11) Range: 7–25	11.17 (4.13) Range: 5–20	26.32 (2)**	Group3, Group2 < Group1	54.67**	Group3 < Group2 < Group1

CCI, Charlson Comorbidity Index. SCC, Subjective Cognitive Complaints. MMSE, MiniMental State Examination. CVLT-SDFR, California Verbal Learning Test, Short Delay Free Recall. CVLT- LDFR, California Verbal Learning Test, Long Delay Free Recall. * p < 0.05, ** p < 0.01.

### Familiarity

GLMMs considering normal response (Gaussian) showed that the best fit model for Familiarity score (see Table 2) was model 2, which includes random effects for the intercepts and slopes, and fixed effects for Evaluation Time [χ^2^(1) = 15.87; *p* < 0.001] and Group [χ^2^(2) = 12.33; *p* < 0.001] but not the effect of the Group × Evaluation time interaction. Neither of the covariates (Age and WAIS-vocabulary score at baseline) or the Evaluation Time × Group interaction were significant.

**TABLE 2:** Summary of models compared for Familiarity.

	Dependent variable: Familiarity		
	Model 1	Model 2	Model 3
Age at baseline	1.283 (1.529)	2.558 (1.556)	2.552 (1.555)
Vocabulary-WAIS	2.453 (1.527)	1.953 (1.549)	1.938 (1.549)
Evaluation Time		4.764*** (1.196)	5.050*** (1.332)
MCI-Stable		1.103 (4.529)	6.311 (8.679)
MCI-Worsened		−18.798*** (5.526)	−19.531 (0.134)
Evaluation Time × MCI-Stable			−2.596 (3.685)
Evaluation Time × MCI-Worsened			0.429 (4.815)
Intercept	221.769*** (1.468)	213.637*** (2.904)	213.064*** (3.134)
Observations	676	676	676
Log Likelihood	−3,186.721	−3,166.436	−3.161.473.160
Akaike Inf. Crit.	6,391.442	6,356.871	6,350.947
Bayesian Inf. Crit.	6,432.048	6,410.959	6,414.007
Bayes Factor	–	37966.384	0.2178

All models include random effects for intercepts and slopes, heteroskedasticity due to the group, and Age and Vocabulary at baseline as covariates. Model 1 is the null mixed model (i.e., intercepts and covariates only); Model 2 is the mixed model with main effects; and Model 3 is the mixed model with main effects and interactions. Coefficients and standard errors (in parentheses). *** p < 0.01.

**FIGURE 2:**
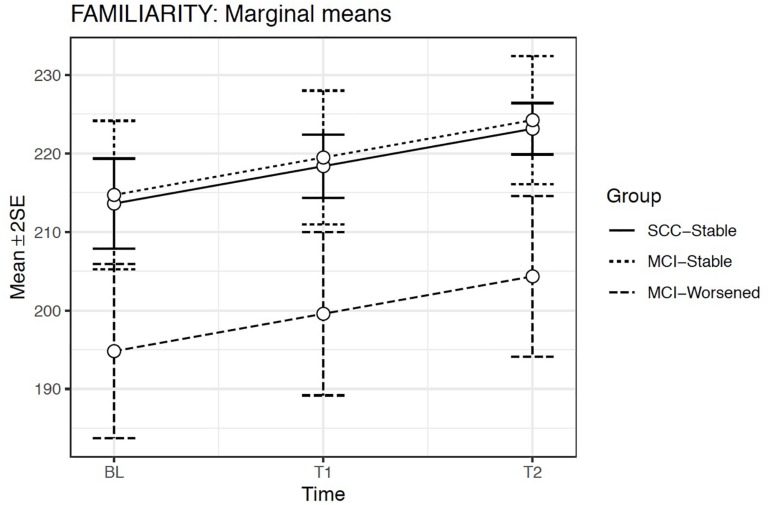
Estimated marginal means and errors bars from Model 2 for Familiarity in the three groups across the three evaluation times. SE, Standard Error; BL, Baseline assessment; T1, Time 1 assessment; T2, Time 2 assessment.

According to this model, the estimated means showed a significant increase in familiarity across the evaluation times in all groups (*p* < 0.001). Familiarity was significantly lower for the MCI-Worsened group than for the SCC-Stable or the MCI-Stable groups at any Evaluation time (*p* < 0.001). The Group × Evaluation time interaction did not reach significance, showing that between-group differences in familiarity were maintained over time (Figure 2).

### Feeling of Knowing

We used GLMMs selecting a Poisson model because of the presence of equi-dispersion. Model 2 provided the best fit (Table 3) and included random effects for the intercepts and fixed effects only for Evaluation time and Group. Model 2 showed a significant effect only for Group [χ^2^(2) = 31.27; *p* < 0.001], but not for Evaluation time or for the Group × Evaluation time interaction. The covariates Age and WAIS-vocabulary score at baseline did not reach significance.

**TABLE 3:** Summary of models compared for Feeling of Knowing.

	Dependent variable: Feeling of Knowing		
	Model 1	Model 2	Model 3
Age at baseline	−0.017*** (0.006)	−0.008 (0.006)	−0.008 (0.006)
Vocabulary-WAIS	0.015** (0.006)	0.010 (0.006)	0.010 (0.006)
Evaluation Time		0.013 (0.007)	0.011 (0.008)
MCI-Stable		−0.017 (0.018)	−0.056 (0.046)
MCI-Worsened		−0.132*** (0.024)	−0.118** (0.060)
Evaluation Time × MCI-Stable			0.020 (0.022)
Evaluation Time × MCI-Worsened			−0.008 (0.031)
Intercept	3.857*** (0.006)	3.845*** (0.015)	3.849*** (0.017)
Observations	751	751	751
Log Likelihood	−1,999.809	−1,981.637	−1,981.159
Akaike Inf. Crit.	4,007.618	3,977.273	3,980.318
Bayesian Inf. Crit.	4,025.470	4,025.514	4,020.485
Bayes Factor	–	4806.453	0.0025

All models included random effects for intercepts and Age and Vocabulary at baseline as covariates. Model 1 is the null mixed model (i.e., intercepts and covariates only); Model 2 is the mixed model with main effects; and Model 3 is the mixed model with main effects and interactions. Coefficients and standard errors (in parentheses). ** p < 0.05, *** p < 0.01.

**FIGURE 3:**
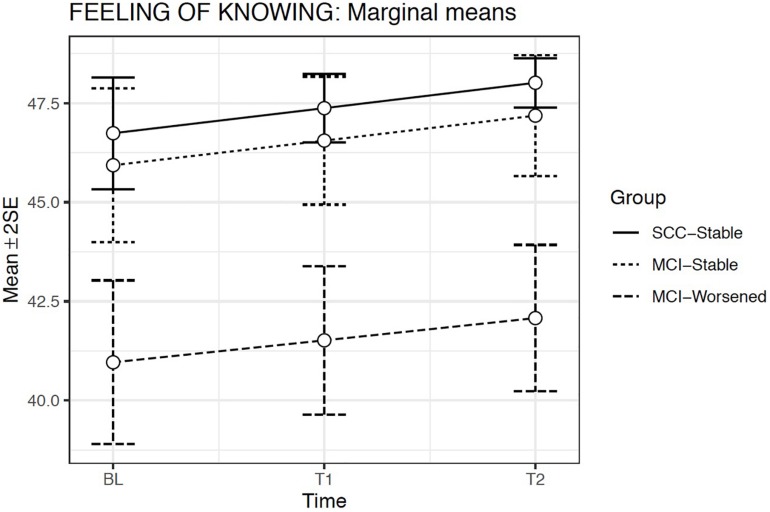
Estimated marginal means and errors bars from Model 2 for Feeling of Knowing in the three groups across the three evaluation times. SE, Standard Error; BL, Baseline assessment; T1, Time 1 assessment; T2, Time 2 assessment.

The estimated means model indicated that Feeling of Knowing scoring was significantly lower for the MCI-Worsened group than for the SCC-Stable or for the MCI-Stable groups at any Evaluation time (*p* < 0.001) (Figure 3).

### ToT Semantic Access

The GLMMs considering normal response (Gaussian) showed that the best fit model was Model 2, which included random intercepts, Evaluation Time [χ^2^(1) = 70.96; *p* < 0.001] and Group effects [χ^2^(2) = 36.88; *p* < 0.001] but not the Group x Evaluation time interaction. The covariates Age at baseline [χ^2^(1) = 4.66; *p* = 0.03] and WAIS-vocabulary score [χ^2^(1) = 9.45; *p* < 0.001] were both significant (Table 4).

**TABLE 4:** Summary of models compared for Semantic Access.

	Dependent variable: Semantic Access percentage		
	Model 1	Model 2	Model 3
Age at baseline	−1.798*** (0.609)	−1.357** (0.629)	−1.335** (0.629)
Vocabulary-WAIS	2.418*** (0.611)	1.943*** (0.632)	1.961*** (0.632)
Evaluation Time		2.234*** (0.265)	2.162*** (0.280)
MCI-Stable		−3.594 (1.835)	−5.578** (2.504)
MCI-Worsened		−15.690*** (2.623)	−11.971** (4.855)
Evaluation Time × MCI-Stable			1.065 (0.913)
Evaluation Time × MCI-Worsened			−2.199 (2.403)
Intercept	91.063*** (0.584)	87.996*** (0.826)	88.129*** (0.843)
Observations	641	641	641
Log Likelihood	−2,273.151	−2,221.715	−2,217.955
Akaike Inf. Crit.	4,560.302	4,463.430	4,459.910
Bayesian Inf. Crit.	4,591.511	4,507.967	4,513.315
Bayes Factor	–	1.38479⋅10^18^	0.069

All models included random effects for intercepts and Age and Vocabulary at baseline as covariates. Model 1 is the null mixed model (i.e. intercepts and covariates only); Model 2 is the mixed model with main effects; and Model 3 is the mixed model with main effects and interactions. Coefficients and standard errors (in parentheses). ** p < 0.05, *** p < 0.01.

**FIGURE 4:**
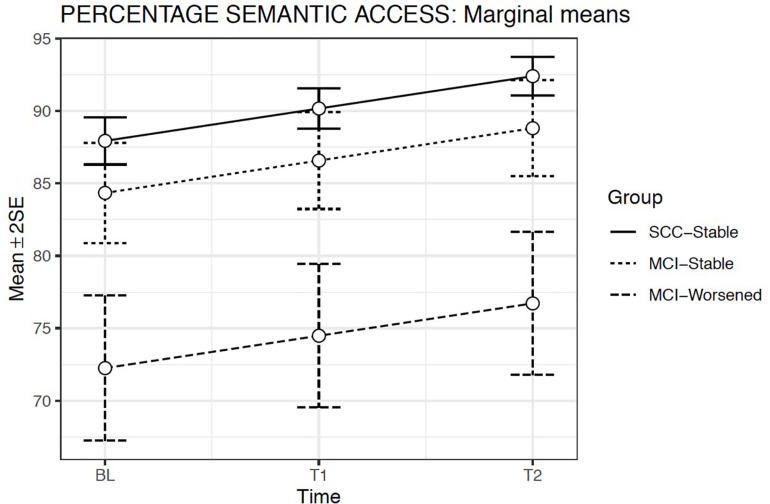
Estimated marginal means and errors bars from Model 2 for Semantic Access in the three groups across the three evaluation times. SE, Standard Error; BL, Baseline assessment; T1, Time 1 assessment; T2, Time 2 assessment.

According to this model, the estimated means showed a significant increase in Semantic access throughout the evaluation times in all groups (*p* < 0.001). Semantic access was significantly lower in the MCI-Worsened group than in the SCC-Stable or the MCI-Stable groups at any Evaluation time (*p* < 0.001). The Group × Evaluation time interaction was not significant, showing that the increase across the Evaluation times in the MCI groups was similar to that observed in the SCC-Stable group (Figure 4).

### ToT Phonological Access

The GLMMs considering normal response (Gaussian) for the proportional measure of Phonological Access showed that Model 2 produced the best fit. This model included random intercepts and slopes, Evaluation Time [χ^2^(1) = 4.56; *p* = 0.03] and Group fixed effects [χ^2^(2) = 30.63; *p* < 0.001] but not the Group × Evaluation time interaction. The covariates Age at baseline [χ^2^(1) = 20.37; *p* = 0.03] and WAIS-vocabulary score [χ^2^(1) = 26.81; *p* < 0.001] were both significant (Table 5).

**TABLE 5:** Summary of models compared for Phonological Access.

	Dependent variable: Phonological Access percentage		
	Model 1	Model 2	Model 3
Age at baseline	−4.946*** (0.854)	−3.769*** (0.835)	−3.742*** (0.835)
Vocabulary-WAIS	5.350*** (0.854)	4.301*** (0.831)	4.332*** (0.830)
Evaluation Time		−0.958** (0.449)	−1.059** (0.485)
MCI-Stable		−11.044 *** (2.573)	−15.663*** (4.171)
MCI-Worsened		−12.160*** (2.898)	−10.437** (4.215)
Evaluation Time × MCI-Stable			2.461 (01.749)
Evaluation Time × MCI-Worsened			−0.987 (1.728)
Intercept	78.651*** (0.830)	82.874*** (1.219)	83.061*** (1.265)
Observations	637	637	637
Log Likelihood	−2,402.963	−2,382.531	−2,378.374
Akaike Inf. Crit.	4,823.925	4,789.062	4,784.747
Bayesian Inf. Crit.	4,863.994	4,842.429	4,846.965
Bayes Factor	–	4.65930445815727e + 47	0

All models included random effects for intercepts and slopes, heteroskedasticity due to the group, and Age and Vocabulary at baseline as covariates. Model 1 is the null mixed model (i.e., intercepts and covariates only); Model 2 is the mixed model with main effects; and Model 3 is the mixed model with main effects and interactions. Coefficients and standard errors (in parentheses). ** p < 0.05, *** p < 0.01.

**FIGURE 5:**
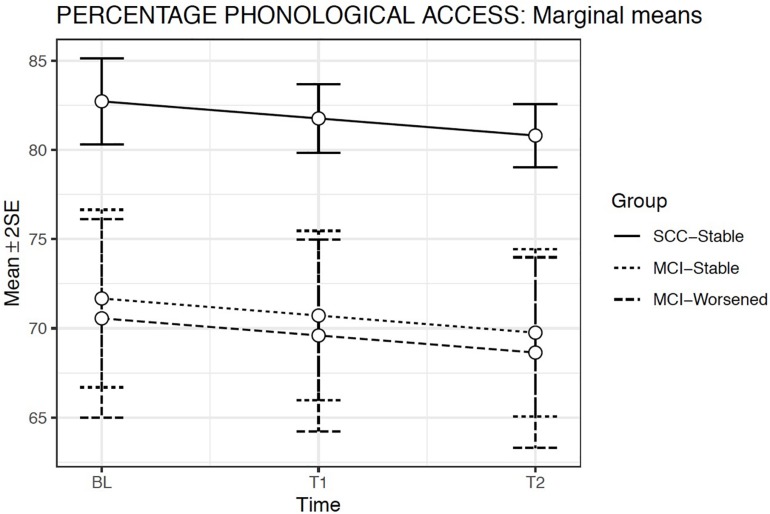
Estimated marginal means and errors bars from Model 2 for Phonological Access in the three groups across the three evaluation times. SE, Standard Error; BL, Baseline assessment; T1, Time 1 assessment; T2, Time 2 assessment.

The two MCI groups showed lower success in phonological access than the SCC-Stable group (*p* < 0.001). The slopes were therefore similar for both MCI groups relative to the reference group (i.e., SCC-Stable) (Figure 5).

### Semantic Fluency

We used GLMMs and selected a model assuming a response according to a negative binomial distribution because of the presence of overdispersion. Model 2 was the best fit model (Table 6) including random effects for the intercepts and fixed effects only for Evaluation time and Group. Model 2 showed a significant effect only for Group [χ^2^(2) = 24.87; *p* < 0.001], but not for Evaluation time or for the Group × Evaluation time interaction. The covariates Age at baseline [χ^2^(1) = 18.70; *p* < 0.001] and WAIS-vocabulary score [χ^2^(1) = 80.24; *p* < 0.001] were both significant (Table 6).

**TABLE 6:** Summary of models compared for Semantic Fluency.

	Dependent variable: Semantic Fluency		
	Model 1	Model 2	Model 3
Age at baseline	−0.088*** (0.016)	−0.068*** (0.016)	−0.067*** (0.016)
Vocabulary-WAIS	0.157*** (0.016)	0.141*** (0.016)	0.142*** (0.830)
Evaluation Time		−0.000 (0.012)	−0.004 (0.013)
MCI-Stable		−0.129 *** (0.046)	−0.116 (0.083)
MCI-Worsened		−0.265*** (0.058)	−0.144 (0.112)
Evaluation Time × MCI-Stable			−0.007 (0.036)
Evaluation Time × MCI-Worsened			−0.068 (0.054)
Intercept	2.774*** (0.015)	2.816*** (0.028)	2.808*** (0.030)
Observations	751	751	751
Log Likelihood	−1,957.598	−1,945.597	−1,944.808
Akaike Inf. Crit.	3,925.196	3,907.195	3.909.617
Bayesian Inf. Crit.	3,947.879	3,943.488	3,954.948
Bayes Factors	–	8.9853	0.0032

All models included random effects for intercepts and slopes, heteroskedasticity due to the group, and Age and Vocabulary at baseline as covariates. Model 1 is the null mixed model (i.e., intercepts and covariates only); Model 2 is the mixed model with main effects; and Model 3 is the mixed model with main effects and interactions. Coefficients and standard errors (in parentheses). *** p < 0.01.

**FIGURE 6:**
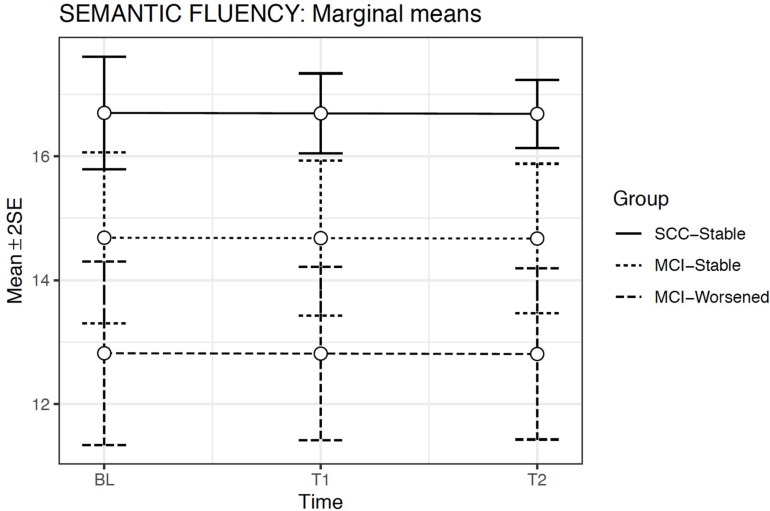
Estimated marginal means and errors bars from Model 2 for Semantic Fluency in the three groups across the three evaluation times. SE, Standard Error; BL, Baseline assessment; T1, Time 1 assessment; T2, Time 2 assessment.

Estimated means model indicated that Semantic Access scores were significantly lower for the MCI-Worsened group than for the SCC-Stable or for the MCI-Stable groups at all Evaluation times (*p* < 0.001) (Figure 6).

## Discussion

The main aim of this study was to analyze the longitudinal patterns of ToT events in patients with MCI according to their changes in diagnostic status, in order to obtain new evidence about the relevance of ToT measures as linguistic markers of MCI. Our findings indicate that ToTs successfully differentiate MCI patients from cognitively unimpaired adults with SCCs at all evaluation times. ToTs seem to occur when the activated semantic representation of a word fails to spread the necessary activation to its corresponding phonological representation. In line with this expectation, our findings show that difficulty in access to phonological representations of proper names is the main index for distinguishing deterioration of lexical access comparing the two stages of the cognitive continuum, SCC and MCI (with the two levels of impairment: stable or worsened). By contrast, greater difficulties in semantic access were only observed in the MCI-worsened relative to the SCC-stable and the MCI-stable groups. Group differences were maintained despite the statistical control for age and level of vocabulary, suggesting no longitudinal influence of the vocabulary on ToTs as indicated in previous cross-sectional studies with cognitively unimpaired old adults (Facal et al., 2012; Salthouse and Mendell, 2013; Shafto et al., 2017). Thus, phonological access seems to be an early lexical marker of post-semantic impairment in the cognitive continuum from SCC to MCI (Juncos-Rabadán et al., 2011, 2014). However, slopes did not change across the follow-ups, and group differences in either phonological or semantic measures of lexical access thus remained stable. This result suggests that the difficulties in phonological or semantic processes in prodromal stage of AD progress similarly, despite the longitudinal stability or deterioration in cognitive status.

Regarding the other ToT measures (familiarity and feeling of knowing), only the MCI worsened group obtained lower scores than the other two groups, indicating greater decline in MCI patients whose cognitive status worsened in relation to the meta-cognitive processes (Schwartz and Metcalfe, 2011). Our findings did not produce evidence that these meta-cognitive measures may be sensitive marker of preclinical and prodromal AD, as they did not show different longitudinal decline patterns according to the diagnostic groups and their stability or progression toward more advance stages of cognitive impairment. They are therefore consistent with the findings of a previous follow-up study (Facal et al., 2016a) although its methodology was not longitudinal (including only baseline and follow-up assessments) and results inform about mean differences between groups; but they contrast with the evidence provided in some cross-sectional studies (Wolk et al., 2013; Pitarque et al., 2016).

Semantic fluency was also significantly more impaired in the MCI worsened patients than in the other two groups. As performance in fluency tasks rely on the successful semantic and phonological processes, this finding further confirms that semantic access is not as good as phonological access in differentiating the stages in the continuum between unimpaired cognition and dementia (Juncos-Rabadán et al., 2013; Vaughan et al., 2018). In addition, semantic fluency remained stable during the evaluation times, and even increased slightly (although not statistically significantly), suggesting that it is not a good predictor of worsening cognitive status.

Longitudinal changes indicated a decline in phonological access over time in all groups, whereas semantic access and familiarity increased and semantic fluency remained stable. These different patterns indicate that semantic access and familiarity were affected by practice effects involved in repetition of the target pictures and names at the successive follow-up evaluations. However, phonological access does not seem to be affected by practice effects, again confirming this measure as a more powerful marker of deficits in lexical access.

We did not observe significant interactions between Group × Evaluation time in any of the measures; longitudinal patterns of increase, decline or stability were therefore similar in all groups, independently of the stage of cognitive impairment. Direct comparison of these findings is not possible, due to the lack of longitudinal research on ToT; however, our findings on semantic fluency are consistent with those reported by Vaughan et al. (2018), who did not find significant differences in semantic fluency (animals) between MCI patients who progressed to AD and MCI non-progressors in a follow-up study with a mean duration of 2.46 years. Pakhomov et al. (2016) also did not find any differences in semantic fluency between MCI and AD participants in a longer longitudinal design, although differences between the cognitively unimpaired group and both MCI and AD groups were reported. We believe that use of participants with subjective complaints (rather than a healthy control group) as the reference group may at least partly explain this inconsistency. Our results are also partly consistent with those of Maruta and Pavao-Martins (2019), who reported a similar rate of decline as in subjects with subjective complaints over time in semantic fluency not related to the follow-up outcome of cognitive impairment. Nevertheless, we must point out some limitations of the present study related to its longitudinal nature and the complexity of diagnostic transitions in MCI. The unbalanced sampling design, even though represents the difference of incidence of SCC and MCI in a naturalistic sample and different rates of stability or progression/worsening, may limit the generalization of the results. Although after visualizing some of the change patterns it might seem reasonable to add non-linear trends in order to improve models fit, incorporating these terms was not possible due to the nature of the current longitudinal study. Specifically, only three measurements were included in the present research and, adding other trends than linear would cause an overfitting problem since there are not enough observations to cope with this complexity. Thus, further research to study other more complex change patterns is required.

In summary, in this study we identified a longitudinal pattern of ToT events in patients with MCI. The findings show that phonological access is impaired in the two groups of MCI participants (those who remain stable, and those who worse) and that performance in phonological access declines over time in all the groups representing cognitive stages prior to dementia. Nevertheless, we must point out some limitations of the present study related to its longitudinal nature and the complexity of diagnostic transitions in MCI. In previous studies, differences in ToT patterns emerged in unidomain and multidomain MCI (Juncos-Rabadán et al., 2013). However, in the present study we considered only two groups of patients with MCI according to changes in symptoms over time. Because of sample size limitations related to diagnostic transitions and attrition (Facal et al., 2015, 2016b), it was not possible to construct different groups according to their cognitive status at baseline or according to patterns of diagnostic evolution. Future studies including different MCI and SCCs subtypes and also with more longitudinal follow-up times may detect group-time interactions, thus allowing the use of Phonological and Semantic access as markers of lexical access, both for cross-sectional differences between diagnostic groups and for longitudinal differences predicting transitions and/or progression to dementia.

## Data Availability Statement

The data supporting the findings of the study are available within the article (Supplementary Material 2).

## Ethics Statement

The studies involving human participants were reviewed and approved by Galician Clinical Research Ethics Committee (Xunta de Galicia, Spain). The patients/participants provided their written informed consent to participate in this study.

## Author Contributions

MC-M, AP, OJ-R, and DF contributed conception and design of the study. DL, AP, and OJ-R were responsible for the methodology. DL performed the statistical analysis. CL-S, SM, MC-M, AN-V, and OJ-R contributed data acquisition and data processing. MC-M, OJ-R, and DF wrote sections of the manuscript. All authors contributed to manuscript revision, read and approved the submitted version.

## Conflict of Interest

The authors declare that the research was conducted in the absence of any commercial or financial relationships that could be construed as a potential conflict of interest.
