# Supplementary material for: Longitudinal Patterns of the Tip-of-the-Tongue Phenomenon in People With Subjective Cognitive Complaints and Mild Cognitive Impairment
Source: Front Psychol. 2020 Mar 13;11:425. doi: 10.3389/fpsyg.2020.00425 (PMC7083146; doi:10.3389/fpsyg.2020.00425)
Supplement: Supplementary file 1 [file Data_Sheet_1.docx]

Additional information of the photographs of the celebrities included in the Tip-of-the-Tongue procedure in the same order of presentation.

| **Name of the celebrity** | **Activity/profession** | **Local/International relevance** |
| --- | --- | --- |
| Alberto Núñez Feijoo | Politician | Local |
| Adolfo Suárez González | Politician | Local |
| Bertín Osborne | Singer | Local |
| Lina Morgan | Comedian | Local |
| Charles, prince of Wales | Monarchy membership | International |
| Miguel Bosé | Singer | Local |
| Hugo Chávez | Politician | International |
| Cristiano Ronaldo | Sportsman | International |
| Cayetana de Alba | Arts personality | Local |
| Felipe VI of Spain | Monarchy membership | Local |
| David Bisbal | Singer | Local |
| Felipe González | Politician | Local |
| Fidel Castro | Politician | International |
| Imanol Arias | Actor | Local |
| Cristina de Bormbón | Monarchy membership | Local |
| Sara Carbonero | Journalist | Local |
| Artur Mas | Politician | Local |
| Queen Elisabeth II | Monarchy membership | International |
| Santiago Segura | Actor | Local |
| Santiago Carrillo | Politician | Local |
| Iñaki Urdangarín | Monarchy membership | Local |
| Julio Iglesias | Singer | Local |
| José María Aznar | Politician | Local |
| Juan Carlos I | Monarchy membership | Local |
| Jordi Pujol | Politician | Local |
| Alfonso Guerra | Politician | Local |
| Elena de Borbón | Monarchy membership | Local |
| Carmen Sevilla | Actress | Local |
| Diana, princess of Wales | Monarchy membership | International |
| Manuel Fraga | Politician | Local |
| Lolita Flores | Singer | Local |
| Esperanza Aguirre | Politician | Local |
| Isabel Preysler | TV Star | Local |
| Letizia Ortiz | Monarchy membership | Local |
| Angela Merkel | Politician | International |
| Mercedes Milá | TV Star | Local |
| Matías Prats | Journalist | Local |
| Montserrat Caballé | Singer | Local |
| Concha Velasco | Actress | Local |
| Barack Obama | Politician | International |
| Pedro Almodóbar | Arts personality | Local |
| Pope Francis / Bergoglio | Pope | International |
| Mariano Rajoy | Politician | Local |
| Pablo Iglesias | Politician | Local |
| María Teresa Campos | TV Star | Local |
| Rafa Nadal | Sportsman | Local |
| Raphael | Singer | Local |
| Queen Sofía | Monarchy membership | Local |
| Vicente del Bosque | Sportsman | Local |
| Rocío Jurado | Singer | Local |
